# Tumour-specific triple-regulated oncolytic herpes virus to target glioma

**DOI:** 10.18632/oncotarget.8637

**Published:** 2016-04-07

**Authors:** Zahid M. Delwar, Guoyu Liu, Yvonne Kuo, Cleo Lee, Luke Bu, Paul S. Rennie, William W. Jia

**Affiliations:** ^1^ Experimental Medicine Program, Department of Medicine, University of British Columbia, Vancouver, Canada; ^2^ Department of Surgery, University of British Columbia, Vancouver, Canada; ^3^ Brain Research Centre, University of British Columbia, Vancouver, Canada; ^4^ Department of Biology, University of British Columbia, Vancouver, Canada; ^5^ Department of Urology, University of British Columbia, Vancouver, Canada

**Keywords:** oncolytic herpes virus, glioma, survivin, microRNA, 5′UTR

## Abstract

Oncolytic herpes simplex virus type 1 (oHSV-1) therapy is an emerging treatment modality that selectively destroys cancer. Here we report use of a glioma specific HSV-1 amplicon virus (SU4-124 HSV-1) to selectively target tumour cells. To achieve transcriptional regulation of the SU4-124 HSV-1 virus, the promoter for the essential HSV-1 gene ICP4 was replaced with a tumour specific survivin promoter. Translational regulation was achieved by incorporating 5 copies of microRNA 124 target sequences into the 3′UTR of the ICP4 gene. Additionally, a 5′UTR of rat fibroblast growth factor -2 was added in front of the viral ICP4 gene open reading frame. Our results confirmed enhanced expression of survivin and eIF4E in different glioma cells and increased micro-RNA124 expression in normal human and mouse brain tissue. SU4-124 HSV-1 had an increased ICP4 expression and virus replication in different glioma cells compared to normal neuronal cells. SU4-124 HSV-1 exerted a strong antitumour effect against a panel of glioma cell lines. Intracranial injection of SU4-124 HSV-1 did not reveal any sign of toxicity on day 15 after the injection. Moreover, a significantly enhanced antitumour effect with the intratumourally injected SU4-124 HSV-1 virus was demonstrated in mice bearing human glioma U87 tumours, whereas viral DNA was almost undetectable in normal organs. Our study indicates that incorporation of multiple cancer-specific regulators in an HSV-1 system significantly enhances both cancer specificity and oncolytic activity.

## INTRODUCTION

Glioblastoma multiforme (GBM) is the most common and aggressive primary brain tumour [1–3]. With the best possible treatment, GBM patients survive for only 12 to 15 months [2]. The current treatment for GBM is limited to surgical resection of the tumour, followed by radiation and chemotherapy [3]. Oncolytic virus (OV) therapy has recently emerged as a promising antitumour therapeutic mainly because its tumour specificity can selectively replicate in tumour cells while sparing normal cells [4]. Among the different oncolytic viruses, oncolytic herpes simplex virus type 1 (oHSV-1) has emerged as one of the most promising OV candidates due to its well-known pathology in humans, extensively researched virology, well-characterized viral genome and its 150kb genome allowing ample space to integrate different transgenes and permitting of specific antiviral therapy as a safety measure [5, 6]. The efficacy and safety of oHSV-1 has been widely investigated and tested in preclinical and clinical glioma models [5].

Viral oncolysis is an important feature of OV since it allows the virus to disseminate inside the tumour mass and to release tumour antigens associated with lytic destruction of tumour cells [7]. This mechanism provides OV therapy with the unique ability to provoke an anti-tumour immune response compared to other tumour vaccines [8]. However, acquiring viruses with a high level of lytic activity while maintaining tumour specificity has always been a challenge. For most oncolytic DNA viruses, the price of tumour specificity is often attenuated viral activity [9]. For instance, in the case of oHSV-1, a common approach to reduce its neurovirulence is to delete the ICP34.5 gene, which significantly attenuates the viral replication efficiency [10, 11].

**Figure 1:**
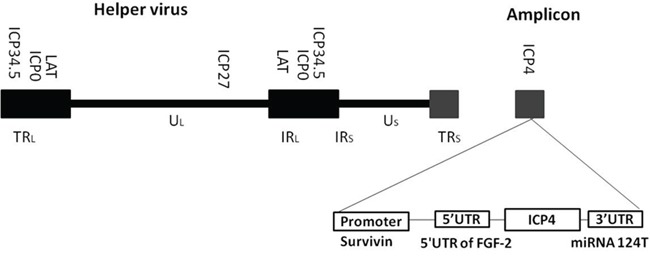
Schematic diagram of cancer-specific triple-regulated HSV-1 amplicon virus (SU4-124 HSV-1) The ICP4 promoter was replaced with the survivin promoter. The 5′UTR of rat fibroblast growth factor -2, and 5 copies of the microRNA 124 target sequence 3′UTR were introduced into the ICP4 gene.

HSV-1 virus replication starts with the expression of immediate early genes, which initiate a cascade of viral gene expression ending in completion of the viral life cycle. Among the 5 essential genes, ICP4 and ICP27 are absolutely required for viral replication and disabling either one or the other results in a non-replicable virus. Previously, we have shown that either ICP4 or ICP27 can be transcriptionally and/or translationally regulated without deleting any other viral genes allowing it to retain tumour specificity in a prostate cancer model [12, 13]. In the present study, we further tested an amplicon/oHSV-1 completed system in which the ICP4 gene is controlled by glioma-specific transcriptional, post-transcriptional and translational triple-regulation in a glioma model. For this virus, the ICP4 gene is transcriptionally controlled by a survivin promoter. In addition, an FGF 5′ UTR region and a 3′UTR containing the target sequence for miR124 are added to regulate expression of ICP4 for tumour-specific translation (Figure 1). Our results showed that this oHSV has enhanced tumour specificity in glioma model.

## RESULTS

### Survivin and eIF4E are overexpressed in glioma cells, but downregulated in normal neuronal cells

**Figure 2:**
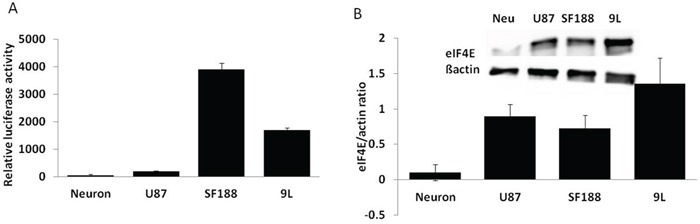
Survivin and eIF4E expression in different cells **A.** The indicated cells were transfected with survivin luciferase and CMV-LacZ reporter plasmids. Total protein was extracted 24 hours post-transfection and subjected to a luciferase and lacZ assay. **B.** Total protein was extracted from the indicated cells and subjected to western blot analysis to measure eIF4E and β-actin expression. Data are presented as means ± SD.

Survivin is well known for its anti apoptotic function and has been reported to be upregulated in many cancers, including gliomas [14–20]. A survivin luciferase reporter assay in rat neurons and various glioma cells confirmed overexpression of the gene in glioma cells (Figure 2A). Upregulation of survivin transcription in glioma cells was also observed by qRT-PCR (data not shown). Since the ICP4 gene was also translationally regulated by the FGF gene 5′ UTR, which requires a high level of eukaryotic translation initiation factor 4 (eIF4E) for successful translation, we performed western blot analysis to measure the eIF4E level in normal neurons and various glioma cells. Our results demonstrated the significantly increased eIF4E expression in glioma cells (Figure 2B), which is consistent with previous results by many other laboratories which showed that eIF4E is overexpressed in many cancers as well as in astrocytic tumours [21–23].

### Micro RNA 124 expression is higher in human and mouse brain tissue

**Figure 3:**
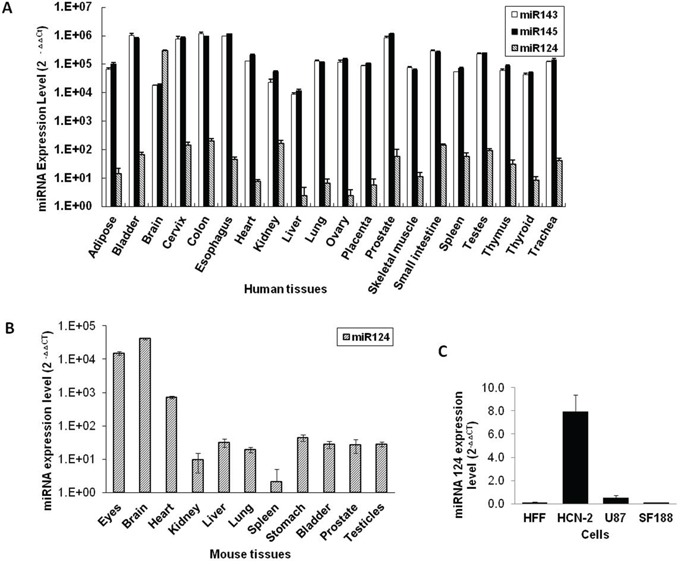
miRNA 124 expression levels in different human and mouse tissues **A.** miRNA 124, 143 and 145 expression levels in different human tissues were measured using a qRT-PCR assay. **B.** The miR124 expression level was measured in tissues extracted from different mouse organs by means of a qRT-PCR assay. **C.** miR124 expression levels in the indicated gliomas and normal cells were detected by qRT-PCR. Data are presented as means ± S.D.

To select an effective micro RNA target for our glioma-specific oncolytic virus, we studied miR124, miR143 and miR145 expression profiles in a panel of different human tissues and found that the miRNA 124 level is significantly higher in human brain tissue (Figure 3A). Moreover, its expression profile in different mouse tissues also confirmed the augmentation of miR124 in the brain (Figure 3B). PCR analysis demonstrated that the mature miR124 level was significantly high in human cortical neurons (HCN-2) compared with human glioma U87 and SF188 cells (Figure 3C).

### miR124 prevented the replication of miRNA regulated virus

**Figure 4:**
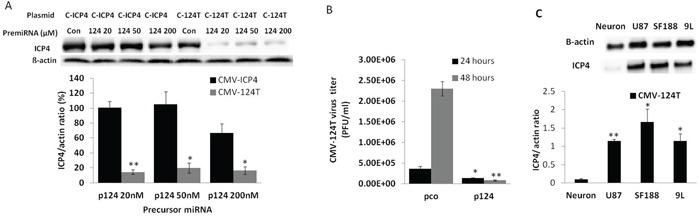
miRNA 124 prevents CMV-124T HSV-1 virus replication **A.** 293FT cells were co-transfected with amplicon plasmid (CMV-ICP4 HSV-1 or CMV-124T HSV-1) and control precursor miRNA or pre-miR 124 at a concentration of 20, 50 or 200 nmol. Total protein was extracted at 48 hours post-transfection. βactin and ICP4 protein levels were measured by western blot analysis. **B.** 293FT cells were transfected with pre-con or pre-miRNA 124. Cells were superinfected with CMV-124T virus at MOI -1 after 48 hours of transfection. Viruses were harvested at 24 and 48 hours post-infection and titrated in Vero cells. Data are presented as means ± S.D. (* P<0.05, ** P<0.01 vs the corresponding control or virus from CMV-ICP4 HSV-1 treated cells) **C.** 124 micro RNA targeted ICP4 protein expression in the indicated cells. Cells were infected with CMV-124 virus at an MOI of 1. Total protein was extracted after 24 hours of treatment. ICP4 expression was measured by western blotting (* P<0.05, ** P<0.01 vs neurons).

To evaluate the specificity of the miR124-regulated ICP4 expression, 293FT cells were co-transfected with different concentrations (20 ng, 50 ng and 200 ng) of miR124 precursor and CMV-124T plasmid. Significantly downregulated ICP4 expression was observed in the presence of miR124 precursor (Figure 4A). Moreover, replication of CMV-124T HSV-1 in which the ICP4 gene is controlled by a 3′UTR region with an miR124 target, drastically decreased in miR124 precursor-transfected cells (Figure 4B). Furthermore, infection with CMV-124T HSV-1 showed that the ICP4 expression was significantly higher in glioma cells (U87, SF188 and 9L) than in primary cultured neurons. (Figure 4C).

### miRNA regulation does not hinder HSV-1 antitumour efficacy

**Figure 5:**
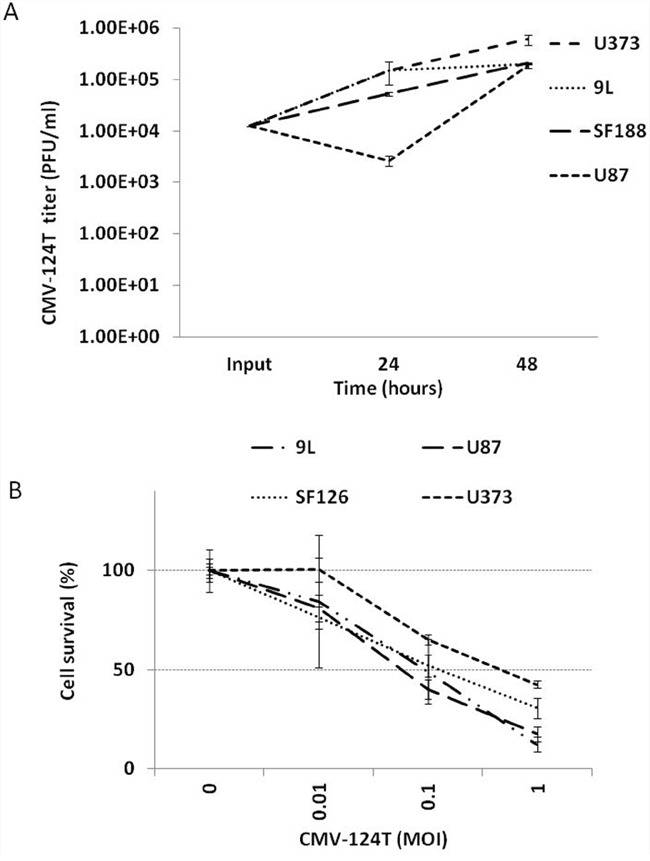
Replication & cytotoxicity of miRNA124 targeted amplicon virus **A.** The indicated cells were infected with CMV-124T HSV-1 at MOI- 0.1. Viruses were harvested at 24 and 48 hours post-infection and titrated in Vero cells. Data are presented on a logarithmic scale **B.** The indicated cells were infected with CMV-124T HSV-1 at the indicated MOIs. After 48 hours of infection, cytotoxicity was measured by an MTT assay.

To determine whether incorporation of the miRNA 124 target sequence in the 3′UTR region of the ICP4 gene would hamper HSV-1 oncolytic activity, we evaluated the replication and cytotoxicity of the CMV-124T virus using a panel of glioma cell lines. A single-step virus growth assay (Figure 5A) and MTT cell proliferation assay (Figure 5B) demonstrated that all glioma cell lines are sensitive to CMV-124T virus oncolysis.

### Survivin promoter, miRNA 124 and 5′UTR triple-regulated amplicon virus (SU4-124 HSV-1) have a strong antitumour effect on glioma cells

**Figure 6:**
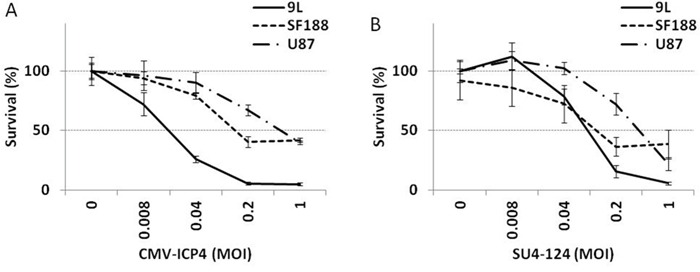
Antiproliferative effect of SU4-124 HSV-1 The indicated cells were infected with either CMV-ICP4 HSV-1 (A) or SU4-124 HSV-1 (B) at the indicated MOIs. Cytotoxicity was measured at 48 hours post-infection by an MTT assay.

Since a high level of miR124 may not only be restricted to neural tissues [24, 25], additional measurement of tumour-specific controls is required to prevent virulence toward non-neural tissues. To that end, we further tested an HSV-1 vector, SU4-124 HSV-1, of which the ICP4 gene is controlled by the survivin promoter and FGF 5′UTR in addition to the miR124 target in the 3′ regions. To evaluate the antitumour effect of the SU4-124 HSV-1 virus, different glioma cells were treated with a non-tumour-specific HSV-1 in which the ICP4 gene was driven by a generic CMV promoter (CMV-ICP4 HSV-1, (Figure 6A) or with the tumour specific SU4-124 HSV-1 for 48 hours (Figure 6B). Inhibitory concentration 50% (IC_50_) values of SU4-124 HSV-1 against 9L, SF188 and U87 glioma cells were around MOI- 0.1, MOI- 0.1 and MOI- 0.05 respectively, while those of CMV-ICP4 HSV-1 against the above cells were approximately MOI-0.02, MOI- 0.1 and MOI- 0.05, respectively.

### Triple-tumour-specific regulation significantly increases tumour specificity

**Figure 7:**
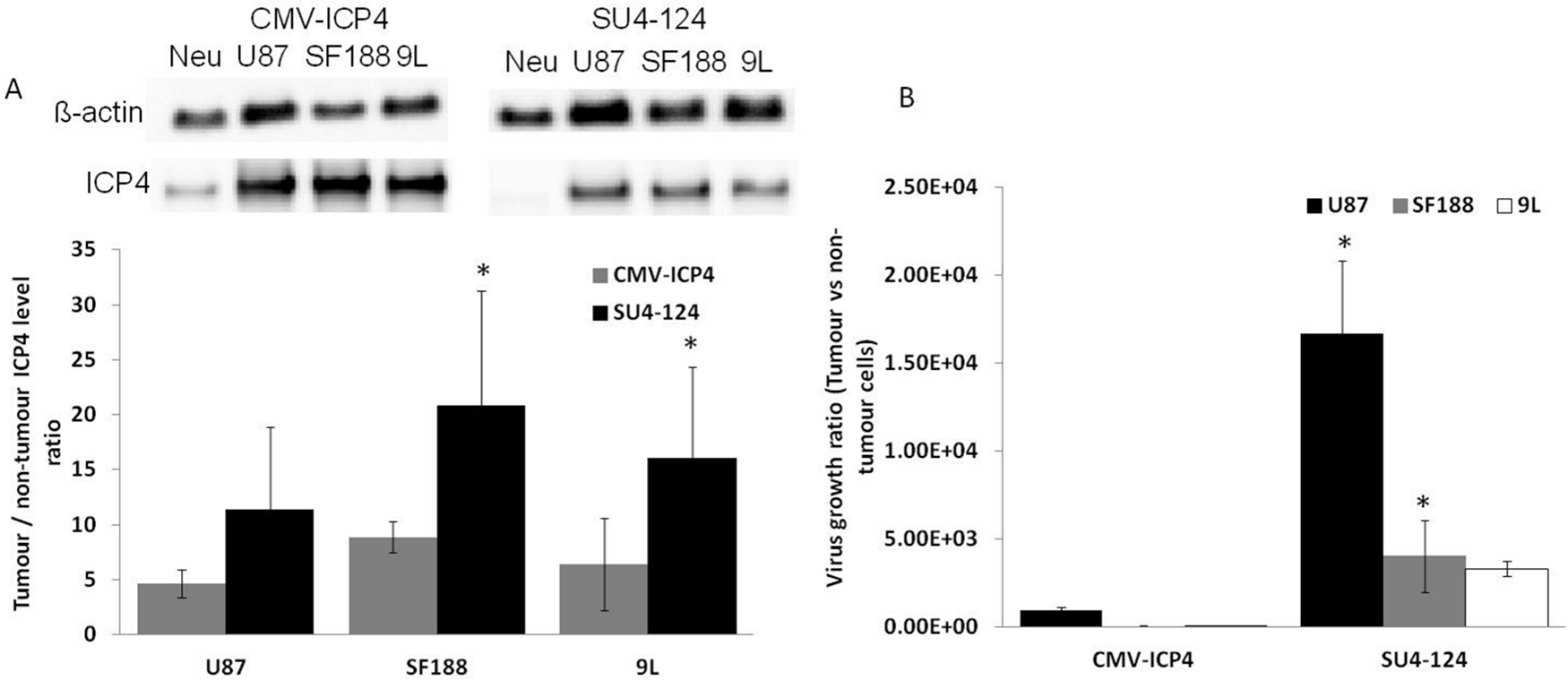
Triple regulation significantly augments tumour specificity **A.** Neurons and different glioma cells were infected with CMV-ICP4 HSV-1 or SU4-124 HSV-1 viruses at an MOI of 1. Total protein was collected 6 hours post infection. ICP4 expression was detected by western blotting. **B.** Neurons and different glioma cells were infected with either CMV-ICP4 HSV-1 or SU4-124 HSV-1 at an MOI of 1. Viruses were harvested at 48 hours post-infection and titrated on Vero cells. Data are presented as means ± S.D., * P<0.05 vs corresponding CMV-ICP4 treatment.

Since the mechanism involved in the glioma specificity of SU4-124 HSV-1 virus depends on glioma-specific expression of the essential viral gene ICP4, we investigated the ICP4 expression level of SU4-124 HSV-1 in normal neurons and glioma cells, as compared with the control CMV-ICP4 HSV-1 virus by western blotting. The ratio of ICP4 expression of glioma cells vs. normal neurons was significantly higher for SU4-124 HSV-1 than that for CMV-ICP4 HSV-1 (Figure 7A). Furthermore, to verify the tumour-specific growth of SU4-124 HSV-1, we evaluated the growth of the SU4-124 HSV-1 and CMV-ICP4 HSV-1 viruses in neurons and various glioma cells. For SU4-124 HSV-1, the ratios of tumour to neurons in viral replication were 16.7, 51.4 and 157 fold higher than those of CMV-ICP4 HSV-1 virus for U87, SF188 and 9L, respectively (Figure 7B).

### SU4-124 HSV-1 virus reduces neuronal toxicity *in vivo*

**Figure 8:**
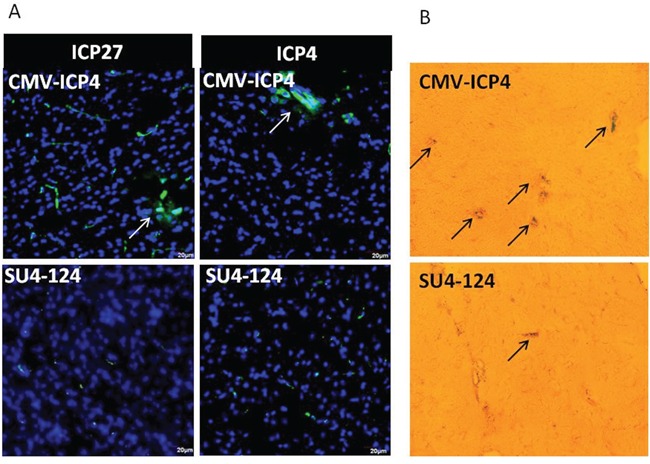
Intracranially injected virus toxicity *in vivo* 2.8X10^5 PFU/ml CMV-ICP4 HSV-1 or SU4-124 HSV-1 amplicon viruses were injected intracranially into C57BL6 mice. Brain tissue was collected at day 15 post-virus injection. **A.** Viral ICP4 and ICP27 expressions were determined by immunohistochemistry of the indicated virus injected brain tissue embedded in OCT and cut into 20-μm sections. **B.** Frozen tissue sections were subjected to X-Gal solutions overnight to detect the helper virus.

To study the neuronal toxicity of the SU4-124 HSV-1 virus, CMV-ICP4 HSV-1 or SU4-124 HSV-1 (2.8X10^5 PFU/ml) were intracranially injected into the brain of C57BL6 mice. Immunostaining for immediate-early viral proteins and virally expressed reporter gene LacZ in the brain showed reduced viral activity in SU4-124 HSV-1-injected mouse brains compared with CMV-ICP4 HSV-1-injected brains (Figure 8A and 8B).

### Tumour-specific triple-regulation enhances the antitumour effect without affecting normal organs *in vivo*

**Figure 9:**
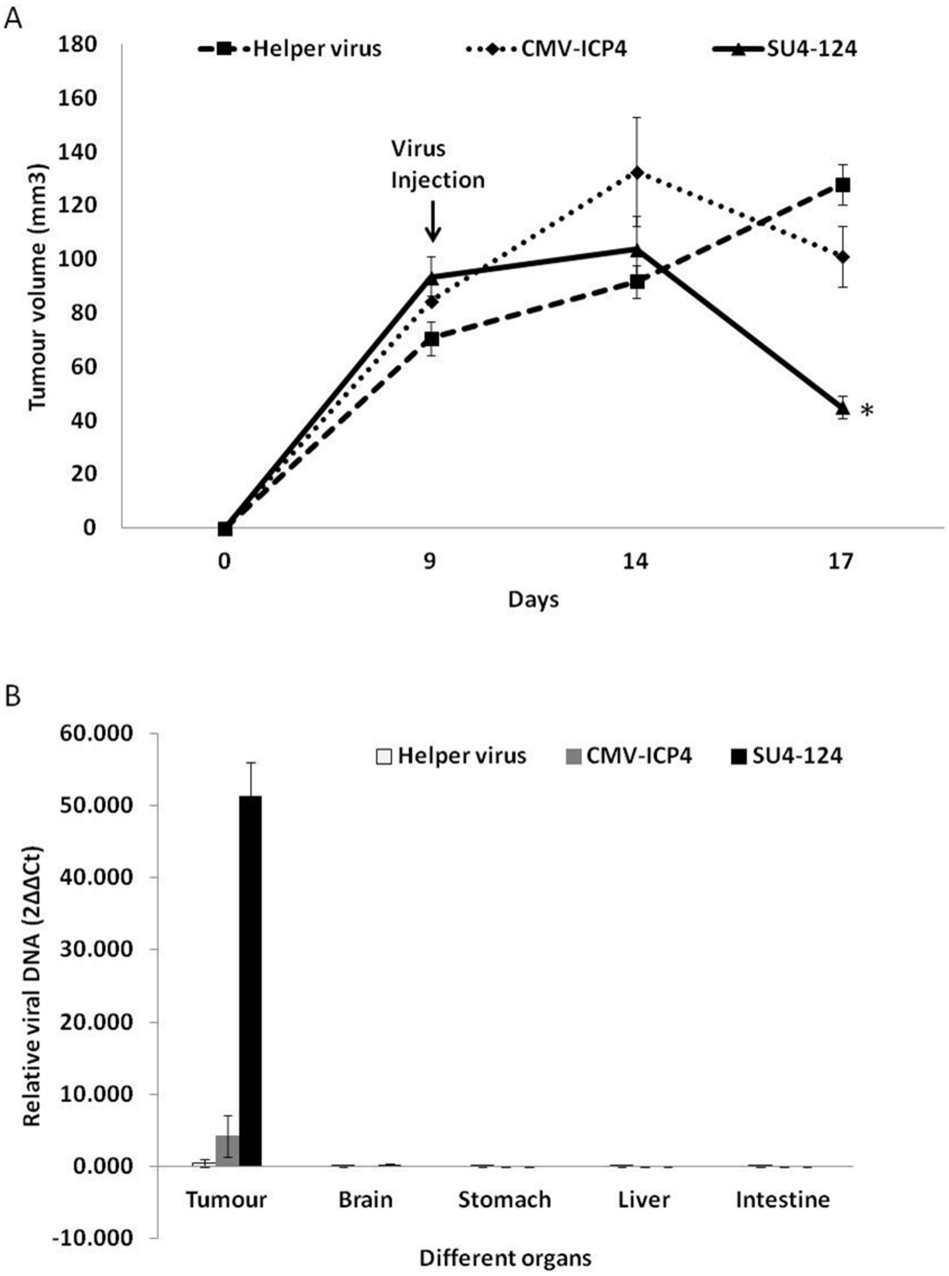
SU4-124 HSV-1 significantly augments the antitumour effect without increasing viral spread to other organs Mice subcutaneously bearing the U87 tumour were intratumourally injected with tumour nonspecific CMV-ICP4 HSV-1 (n=5; 2.4X10^7 PFU/ml helper : 6X10^6 PFU/ml amplicon) or tumour-specific SU4-124 HSV-1 (n=5; 2.4X10^7 PFU/ml helper : 6X10^6 PFU/ml amplicon) or ICP4^−−^ 3galΔ3 (n=5; 2.4X10^7 PFU/ml helper only) at a 4:1 helper : amplicon ratio. **A.** The tumour volumes were measured using calipers [(height X length X width)/2)]. Data are presented as means ± S.E, * P<0.001, SU4-124 vs helper virus treatment. **B.** Total genomic DNA was extracted from the different harvested organs of the indicated mice treated with virus (n=2). Viral genomic DNA (ICP27) was detected by qPCR and normalized to β-actin.

To evaluate the antitumour efficacy of the SU4-124 HSV-1 virus, subcutaneously implanted U87 tumours were treated with either SU4-124 HSV-1 (2.4X10^7 PFU/ml helper : 6X10^6 PFU/ml amplicon) or CMV-ICP4 HSV-1 (2.4X10^7 PFU/ml helper : 6X10^6 PFU/ml amplicon), or with ICP4- helper virus (2.4X10^7 PFU/ml helper) only by intratumoural injection. Both CMV-ICP4 HSV-1 and SU4-124 HSV-1 caused significant tumour regression at 8 days post injection (Figure 9A). Animals were euthanized at day 8 post virus injection (day 17 post tumour implantation) to harvest the tumour and to determine copy number of viral genome by qPCR in the tumour to confirm lytic viral replication inside of the tumours (Figure 9B). Meanwhile, SU4-124 HSV-1 injected tumours showed 2.25 fold greater regression than CMV-ICP4 HSV-1 injected mice at day 8 post virus injection (Figure 9A). In addition, a 12.1-fold increase in viral genome copies was seen in SU4-124 HSV-1-injected tumours compared to those injected with CMV-ICP4 HSV-1 (Figure 9B).

## DISCUSSION

In this study, we developed a triple-regulated oncolytic amplicon viral system that generates tumour-specific oncolysis with a replication-defective helper virus. Reduced toxicity and the enhanced oncolytic potency compared to the wild type virus were also observed.

Certain microRNAs are differentially expressed in tumour and nontumour cells [26]. We previously showed that inclusion of miRNA 143 and 145 target sequences into the 3′UTR of a HSV-1 essential gene restricted viral replication in prostate cancer cells [13]. In the present study, we confirmed that the miRNA 124 level is very high in human and mouse brain tissues as reported in several previous studies [27–29]. Therefore, miRNA 124 may be a better choice for targeting GBM than miRNA 143 & 145. Here, we also observed that miRNA 124-targeted oHSV-1 (CMV-124T HSV-1) expresses ICP4 in various glioma cells but not in normal neuronal cells. In addition, CMV-124T HSV-1 virus-replication-mediated oncolysis was observed in a panel of glioma cells. However, the virus replication and ICP4 protein expression was completely abolished in the presence of precursor miRNA 124.

Since enhanced tumour specificity often comes at a price, namely reduced viral oncolytic activity [9, 11], we attempted to circumvent this disadvantage by using a tumour specific promoter that is highly expressed in glioma cells. Interestingly, the survivin promoter gave rise to transcriptional activity that was higher than CMV and even higher than the native HSV-1 ICP4 promoter. Our results showed that combined with 5′UTR and 3′UTR miR124 translational regulators, survivin promoter-driven ICP4 expression was higher in tumour cells.

Oncolytic virotherapy is mainly used to specifically deplete tumour cells with replication capable viruses through oncolysis: a cell killing mechanism characterized by the lysis of tumour cells through the course of virus replication [4]. However, it has been accepted that there are probably two major mechanisms for the anti-tumour effects by oncolytic viruses. One is by virally induced cell killing through cell lysis [4] and apoptosis [30]; the other is virally induced host immune response [31]. In the present study, athymic nude mice were used to develop human glioma mouse model [32], which lacks T-lymphocytes but the innate immune system is partially functional [33]. However, SU4-124 HSV-1 caused tumour regression is likely due to viral lysis and other virally induced direct cell death as the nonreplicable helper virus, which may stimulate similar innate immune response equally effective as the replicable HSV-1 [34], did not show the same efficacy in tumour regression. Agreeable to this notion, it seems that the tumour regression was related to the efficiency of intratumoural viral replication as the CMV-ICP4 HSV-1 virus that had less viral replication rate in the tumour also showed less efficacy in tumour regression compared to that of SU4-124 HSV-1.

Another feature of this study was that we used an amplicon expressing the essential viral gene to supplement a defective helper HSV-1 for oncolytic viral replication. Considering that HSV-1 may establish latency in the brain after treating the glioma, an amplicon dependent oncolytic virus system may be safer clinically than conventional oncolytic HSV-1 since the amplicons are plasmid constructs that are unlikely to persist in cells, which renders replication and reactivation of the helper virus virtually impossible. A potential concern in using an amplicon-supplemented oHSV system is the need to maintain a consistent ratio of helper to amplicon. This was solved in our study by using an essential viral gene carried by the amplicon to supplement the helper virus. Our study showed that, with this design, the ratio of amplicon to helper is always kept below 1 (Figure S1), which means that the pure helper virus can always be used to dilute the mixture and maintain a consistent ratio. Thus, this study has shown for the first time that a triple-regulated ICP4 gene expressed from an amplicon can be used to supplement a replication-defective HSV-1 to reduce toxicity and enhance oncolysis to destroy glioma.

## MATERIALS AND METHODS

### Cell culture

Human glioma cell lines (U87, U373, SF188, SF126 a rat glioma cell line (9L), human embryonic kidney cells (293FT) and a monkey kidney cell line (vero) and 7B (ICP4 and ICP27 expressing cells) were maintained in Dulbecco's modified Eagle's medium (DMEM) supplemented with 10% fetal bovine serum and a 1% antibiotic mixture (penicillin and streptomycin). The human cortical neuron cell line (HCN-2) used was purchased from ATCC (Manassas, USA) and maintained in DMEM medium containing 4 mM L-glutamine, 4500 mg/L glucose, 1 mM sodium pyruvate, and 1500 mg/L sodium bicarbonate (ATCC, USA), supplemented with 10% fetal bovine serum. Primary rat neurons were cultured in neurobasal medium supplemented with B27 and 5 mM glutamax (Invitrogen, Canada). Human fibroblast (HFF) cells were maintained in MEM (Invitrogen, Canada) medium supplemented with 10% fatal bovine serum. All cultures were maintained at 37°C in 5% CO_2_.

### Plasmid constructs

CMV-ICP4 HSV-1 amplicon plasmid construction was described by Cleo et al. [35]. CMV-124T HSV-1 plasmid was constructed by inserting five tandem copies of miRNA 124 complementary sequences (5**’-CTCGAGCGGTTAATTAACGGGCATTCACC-GCGTGCCTTACGATGGCATTCACCGCGTGCCTTAGATCGGCATTCACCGCGTGCCTTAGTCAGGCATTCACCGCGTGCCTTAGTCAGGCATTCACCGCGTGCCTTAGCTATCGATGCAGT-3′**) into the 3′ end of the ICP4 gene before the poly A signal in CMV-ICP4 HSV-1 plasmid. SU4-124 HSV-1 plasmid was constructed by replacing the CMV promoter of CMV-124T HSV-1 plasmid with a 268 bp survivin promoter [36], upstream to a rat FGF 5′UTR (Figure 1) [12]. All plasmids were constructed using a previously described protocol [12, 13, 35].

### Amplicon virus preparation

To prepare the amplicon viruses, corresponding plasmids were transfected into Vero or U87 cells by using Lipofectamine 2000 transfection reagent (Invitrogen, Canada) according to the manufacturer's instructions. After 48 hours, transfected cells were superinfected with helper (3galΔ3) virus at MOI-2. At 4 to 5 days post superinfection, amplicon viruses were harvested and amplified on SF188 glioma cells. Helper virus was amplified on 7B cells. Viruses were then subjected to titration on Vero and 7B cells to measure the concentration of amplicon and helper respectively, as described previously [13, 35].

### Virus replication assay

Cells plated on a 24-well plate were infected with the viruses at a multiplicity of infection (MOI) of 0.1 or 1. Virus infection and treatment were carried out in DMEM supplemented with 10% FBS and 1% antibiotics. At 2-3 days post-infection, viruses were harvested and freeze thawed three times and then titrated in triplicate on Vero cells by a standard plaque assay on 12-well plates.

### Precursor miRNA transfection

293FT cells were co-transfected with amplicon plasmid (CMV-ICP4 HSV-1 or CMV-124T HSV-1) at a concentration of 200 ng and precursor miRNA (control pre- miRNA or pre-miR 124) at a concentration of 20, 50 or 200 nmol using Lipofectamine 2000 transfection reagent (Invitrogen, Canada) according to the manufacturer's instructions.

### Luciferase reporter assay

293FT cells were co-transfected with survivin luciferin reporter plasmid (1.2 μg) and CMV-LacZ reporter plasmid (0.3 μg) using Lipofectamine 2000 transfection reagent (Invitrogen, Canada) according to the manufacturer's instructions. Total protein was extracted at 24 hours post transfection and subjected to luciferase and β-galactosidase detection using a luciferase assay kit (Promega, Canada) and a β-galactosidase enzyme assay kit (Promega, Canada) respectively, according to the manufacturer's instructions.

### Real time PCR

Total genomic DNA from tumours and various organs were extracted using an EZNA tissue DNA kit (Omega bio-tek). The viral ICP27 copy number was obtained by quantitative real time PCR (Quantstudio 6 Flex qPCR apparatus, Applied Biosystems) using 5′-GTCTGGCGGACATTAAGGACA-3′ (forward) and 5′-TGGCCAGAATGACAAACACG-3′ (reverse) primers. β-actin was used as an endogenous control. 10 ng of DNA were added to a 25 μl master mix of SYBR green (Invitrogen, Canada) supplemented with forward and reverse primers. Total RNA from different human tissues were purchased from Ambion, Canada. Total RNA from different mouse tissues and cell lines were extracted using Triozol (Invitrogen, Canada) according to the manufacturer's protocol and miRNA 124, 143 and 145 from tissue extracted RNA (Figure 3A & 3B) were detected using a previously described method [13]. miRNA 124 in HCN-2, HFF and glioma cells (Figure 3C) was detected with a miScript PCR Starter kit and an miR124 miScript Primer Assay, following the manufacturer's protocol (Qiagen, Canada).

### Cytotoxicity assay

Cells were seeded in a 96-well plate at a density of 1 × 10^4^ and allowed to settle overnight. They were then treated with vehicle only or viruses with different MOIs. Cell viability was measured after 2 or 3 days of treatment by means of an MTT assay (Sigma, Canada) according to the manufacturer's instructions. In brief, cells were incubated with MTT solution for 3 hours at 37°C and then incubated with lysis buffer overnight. The next day, cell viability was measured at 595 nm by using a plate reader (Envision 2103 Multilabel Reader, Perkin Elmer).

### Western blots

Total protein was extracted from cultured cells by using sample buffer (125 mM Tris-HCL, 50% glycerol, 4% bromophenol blue and 5% 2-mercaptoethanol). Proteins were boiled in a heat block for 5 minutes, subjected to SDS-PAGE (8% gel), transferred to nitrocellulose membranes and then blocked in 5% nonfat milk (Bio-Rad) in TBS-Tween 20 (TBS-T) for 1 hour at room temperature. The membranes were then incubated overnight at 4°C with either anti-eIF4E antibody (1:1000; Cell Signalling, Danvers, MA), anti-ICP4 antibody (1:750; Abcam, Cambridge, MA), anti-ICP27 antibody (1:1000; Abcam, Cambridge, MA) or anti-β-actin antibody (1:1000; Cell Signaling, Danvers, MA). Then next day, the membranes were washed with TBS-T three times and incubated with the corresponding secondary antibodies (1:3000; Perkim Elmer, Boston, MA) for 1 hour at room temperature. After washing three times with TBS-T, membranes were visualized using ECL reagent (Perkim Elmer, Boston, MA) and a VersaDoc imaging system (Bio-Rad) and band densities were then measured using ImageJ software (NIH, Bethesda, MD).

### Immunohistochemistry

Harvested tissues were fixed with 4% paraformaldehyde for 24 hours and then incubated with 30% sucrose for 72 hours. Tissues were then embedded in OCT (Sakura Tissue-Tek), sectioned (20 um) using a cryostat (Leica CM 3050 S), prepared on Fisherbrand™ Superfrost™ Plus microscope slides (Fisher Scientific, Canada) and stored at -80C. During immunostaining, frozen sections were washed with PBS, and incubated with 3% albumin bovine serum (ABS) dissolved in PBS containing 0.1% Triton X-100 for one hour to block unspecific binding. Cells were then incubated overnight with either anti-ICP4 antibody (1:200; Abcam, Cambridge, MA) or anti-ICP27 antibody (1:200; Abcam, Cambridge, MA) diluted in PBS containing 0.1% Triton X-100 solution at 4°C. The next day after washing 3 times with PBS, sections were incubated with goat anti-mouse IgG Alexa Fluor 488 secondary antibody (1:500; Invitrogen, Canada) for one hour at room temperature. After incubation with secondary antibody, sections were washed 3 times with PBS and mounted with Dapi Fluoromount G (Electron Microscopy Sciences). Sections were then visualized and imaged using a confocal microscope (Olympus, Canada).

### Intracranial virus toxicity assay

Female C57BL6 mice were purchased from Harlan Laboratories. CMV-ICP4 HSV-1 or SU4-124 HSV-1 viruses were injected intracranially using a stereotactic frame. Animals were monitored on a daily basis to record any sign of toxicity. At 15 days after the virus injection, the animals were euthanized by CO2 asphyxiation. Then the virus-treated brain tissues were harvested and subjected to cryostat sectioning.

### β-galactosidase staining

Frozen-fixed tissue sections were washed twice with PBS and then incubated with 1 mg/ml X-Gal solution (Sigma, Canada) diluted with X-Gal staining solution (5 mM K_3_Fe, 5 mM K_4_Fe and 2 mM MgCl_2_) at 37°C overnight. Cells were then counterstained with 1% eosin. Stained cells were visualized and imaged by using a light microscope.

### U87 xenograft mouse model

Female athymic nude mice were purchased from Harlan laboratories. 2.5X10^6 human glioma U87 cells in 100 ul of PBS containing 0.75 mg Basement Membrane Extract (VWR, Canada) were subcutaneously implanted into the left flank. When the tumour size reached ∼100 mm3, mice were administered a single dose of 3galΔ3, or CMV-ICP4 HSV-1 or SU4-124 HSV-1 virus by intratumoural injection. Tumour volume was measured at different time points using calipers (Height X Length X Width/2). At the end of the experiment, mice were euthanized by CO2 asphyxiation and tumours as well as several organs (brain, stomach, intestine, liver) were harvested. All in-vivo experimental procedures were approved by the UBC Animal Care Committee and performed according to the guidelines of the Canadian Council on Animal Care.

### Statistical analysis

Statistical analysis was performed by using Microsoft Excel and a significance P<0.001, P<0.01, or P<0.05 was determined using a 2 tailed Student's t-test. All data are expressed as means ± SD or ± SE.

## SUPPLEMENTARY FIGURES


