# Supplementary material for: Tumour-specific triple-regulated oncolytic herpes virus to target glioma
Source: Oncotarget. 2016 Apr 7;7(19):28658–69. doi: 10.18632/oncotarget.8637 (PMC5053753; doi:10.18632/oncotarget.8637)
Supplement: Supplementary file 1 [file oncotarget-07-28658-s001.pdf]

Tumour-specific triple-regulated oncolytic herpes virus to target glioma

SUPPLEMENTARY FIGURE

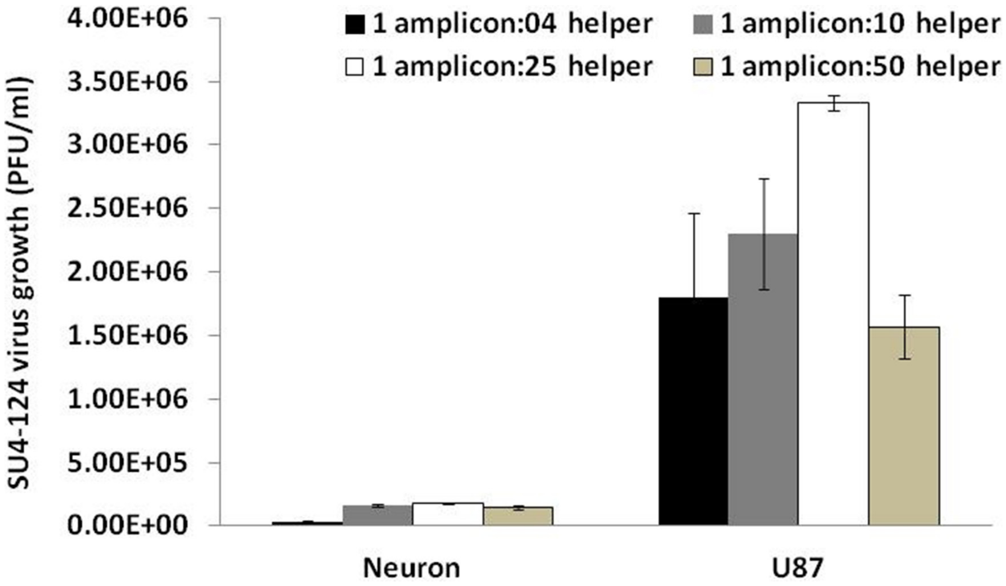

Supplementary Figure S1: Effect of the amplicon and helper ratio on tumour-specific growth. Since amplicon virus is a combination of helper virus and the amplicon, we evaluated the replication of different amplicon and helper ratio combinations in neurons and U87 cells. Both types of cells were infected using the indicated amplicon and helper (MOI-1) ratio of SU4-124 HSV-1, were harvested at 72 hours post-infection and were then titered on Vero cells. We observed a 54, 14.3, 18.2 and 10.7 fold decrease in the titer of neurons compared to U87 cells at an amplicon : helper ratio of 1:4, 1:10, 1:25 and 1:50, respectively (Figure S1).
